# How It Started...How It Is Going: From All-Polymer Batteries to Living Fibers−A Journey through Biobased Innovation

**DOI:** 10.1021/acs.nanolett.6c00054

**Published:** 2026-03-19

**Authors:** Gustav Nyström

**Affiliations:** † Cellulose and Wood Materials Laboratory, 28501 Empa − Swiss Federal Laboratories for Materials Science and Technology, Überlandstrasse 129, 8600 Dübendorf, Switzerland

That first paper did more than just share data with the scientific community; it helped shape my interdisciplinary focus and the unconventional nature of the research we conduct today.

Looking back at my early days as a Ph.D student, I remember the excitement of submitting my first academic paper. At that time, I was working in the lab of Prof. Maria Strömme at Uppsala University, where I studied the templated polymerization of conducting polymers (polypyrrole) on the surface of cellulose nanofibrils. I recall my fascination of being able to chemically synthesize a polymeric material that could efficiently both conduct electricity and transport ions. Using transmission electron microscopy (TEM), we discovered individual cellulose nanofibrils covered with a polypyrrole layer in the 10s of nanometer range. We hypothesized that polymer templating on the surface of the cellulose nanofibrils allowed us to align the conducting polymer chains, which led to optimized electronic conductivity while thin layers enabled rapid ionic transport. This fascinating fibrillar structure of the conductive nanofibrils also allowed us to process macroscale materials in the forms of hydrogels and paper-like membranes.

We were particularly excited about the potential to rapidly electrochemically oxidize and reduce these materials, which we demonstrated in an all-polymer battery−or more precisely a supercapacitor−relying on the pseudocapacitive behavior of polypyrrole. The thrill of getting these results published in *Nano Letters*, a premier research outlet for nanotechnology, nanoscale, and interdisciplinary materials science, as my first academic paper was an unforgettable milestone in my career.^1^

After that first publication, my career unfolded in ways I could never have predicted. I found myself increasingly drawn into the world of biobased nanomaterials, moving from fundamental nanoscale research to real-life applications focused on sustainability. After completing my Ph.D, I joined Prof. Lars Wågberg at KTH Royal Institute of Technology as a postdoc. His lab was dedicated to understanding the potential of well-ordered colloidal nanofibril properties. This was an incredibly exciting time as I got to delve into the colloidal properties of nanocellulose and layer-by-layer self-assembly. We explored the kinetic arrest of differently charged cellulose nanocrystals and cellulose nanofibrils in colloidal glasses and gels, as well as the early lateral formation of liquid crystal phases.^2,3^

The work on layer-by-layer self-assembly complemented my previous experience in electronic materials. We introduced surface-functionalized carbon nanotubes within the assembled multilayers, enabling electronic and electrochemical functionality in the materials. This research ultimately led to successful layer-by-layer assembly of energy storage devices within complex cellulose foam frameworks.^4^

I then continued my postdoctoral training at ETH Zurich, joining the lab of Prof. Raffaele Mezzenga to study the structure and chirality of single cellulose and protein nanofibrils, their liquid crystal self-assembly, and their applications in functional materials. Coming from the nanocellulose community, it was rewarding to learn about the similarities and potential synergies offered by the amyloid fibril system. The former (nanocellulose) system is derived top down using physical and chemical methods. This renders rigid, high aspect ratio colloids typically bearing a single charged group. The latter (amyloid) system is instead a bottom-up supramolecular assembly of semiflexible fibrils bearing a multitude of functional groups and specific material affinities dictated by the native protein composition.

Using high-resolution atomic force microscopy (AFM) coupled with statistical analysis, we were able to directly observe the single fibril right-handed chirality within the nanocellulose system. This observation was contrasting with the typical left-handed chirality of the amyloid fibrils.^5^ This finding established a direct relationship between the right-handed chirality of individual nanocellulose fibrils and the left-handed chirality of the chiral nematic liquid crystal phase.^6^

From the nanocellulose field we knew that shorter rod-like cellulose nanocrystals form stable chiral nematic phases while longer kinked cellulose nanofibrils are trapped in gel or glass-like phases. Leveraging this knowledge, we began experimenting with the physical fragmentation of the amyloids into shorter fibrils using sonication or hydrodynamic shearing. With these shorter amyloid fibrils at hand, we were then able to drive their lyotropic liquid crystal assembly and were very excited to identify, for the first time, the amyloid chiral nematic phase. The observed chirality transfer of the amyloid fibrils was reversed compared to nanocellulose, this time from left-handed fibril to right-handed cholesteric.^7^

Building on the distinct interactions between the amyloids and inorganics, we also explored the use of amyloids as templates for functional materials similarly to the first *Nano Letters* paper. We synthesized a new class of ultraporous gold aerogels with tunable gold particle size, shape, and composition, demonstrating solid gold analogues with up to 3 orders of magnitude less weight than conventional gold alloys, along with additional catalytic and sensing functionalities.^8^

These experiences, from fundamental understanding of biobased colloidal self-assembly to the transfer of material functionalities from the nano to the macroscopic scale, have profoundly influenced my current research. Today I lead my own research lab at the Swiss Federal Laboratories for Materials Science and Technology (Empa). The work we conduct still focuses on biobased building blocks and multiscale material structures with an emphasis on improved sustainability, maximized resource utilization, and tailored embedded material functionalities. Our team operates at the intersection of materials science, nanotechnology, biology, and sustainable-centric innovation—topics that all align with the interdisciplinary profile of *Nano Letters*.

Continuing the research trajectory initiated by that first academic paper on conducting polymer supercapacitors, we have demonstrated fully 3D printed supercapacitors,^9^ water-activated paper batteries,^10^ and fungal biobatteries.^11^ These are all energy storage systems utilizing biobased and biodegradable materials as active components without sacrificing energy storage performance. Additionally, we extensively work on incorporating living microorganisms such as fungi into materials and applications that allow replacement of fossil-based components. A recent highlight is the discovery of living fibers produced from fungal biomass, which combine the advantages of colloidal fiber-based material processing with the dynamic functionalities of a living material.^12^ This allows for the production of active living emulsions that increase stability over time and films where the living component can tune the mechanical properties as desired. These systems are not only biodegradable but can also take on an active function as biodegrading materials.

Reflecting on this journey, I am first and foremost deeply thankful to my mentors and scientific collaborators. Second, I am thankful to the editors of *Nano Letters*−that first paper did more than just share data with the scientific community; it helped shape my interdisciplinary focus and the unconventional nature of the research we conduct today.
